# Ecology of cryptic invasions: latitudinal segregation among *Watersipora* (Bryozoa) species

**DOI:** 10.1038/srep00871

**Published:** 2012-11-28

**Authors:** Joshua A. Mackie, John A. Darling, Jonathan B. Geller

**Affiliations:** 1 Moss Landing Marine Laboratories, 8272 Moss Landing Road, Moss Landing, CA 95039, USA; 2 National Exposure Research Laboratory, US Environmental Protection Agency, 109 T. W. Alexander Drive, Research Triangle Park, NC 27711, USA; 3 Current address: Department of Biological Science, San José State University, CA 95192-0100, USA

## Abstract

*Watersipora* is an invasive genus of bryozoans, easily dispersed by fouled vessels. We examined Cytochrome c oxidase subunit I haplotypes from introduced populations on the US Pacific coastline to investigate geographic segregation of species and/or haplotypes. In California, the *W. subtorquata* group fell into three major sub-groups: *W. subtorquata* clades A and B, and *W*. “*new sp*.”. *W. subtorquata* clades A and B were common in southern California south of Point Conception, a recognized biogeographic boundary, whereas further north, *W. subtorquata* clade A and *W*. *n*. sp. were frequent. The southern California region also had colonies of a morphologically distinct species, *W. arcuata*, also found in southern Australia and Hawaii; COI variation indicates a common ancestral source(s) in these introductions. The distribution of *Watersipora*-complex lineages on different coastlines is shown to be temperature correlated. Accordingly, pre-exisitng temperature-based adaptations may play a key role in determining invasion patterns.

Genetic studies have widely supported the appearance of ‘cryptic’ evolutionary divergence at multiple levels of the taxonomic hierarchy, (e.g. multiple species existing within taxa traditionally considered to be single species^1^,^2^,^3^). The non-native establishment of such diversity is typically referred to as ‘cryptic invasion’^4^, and these phenomena often reflect unrecognized multiple introduction events. Given the unsettled taxonomic state and the high rate of transport by anthropogenic vectors for these taxa, it is not surprising that processes underlying marine invasions remain poorly understood^4^,^5^,^6^. For example, there are few cases in which one could confidently determine from vector history alone, in the absence of genetic information, whether the invasive spread of an organism is the result of propagules from one area or multiple areas^4^.

The existence of cryptic genetic diversity suggests the possibility that corresponding ecological differences could be important for patterns of invasion^7^. Limited examples suggest that invasion potential among such lineages can vary widely, presumably reflecting intrinsic ecological differences. For example, mussels in the genus *Mytilus* include three cryptic species with different environmental tolerances, of which the warmer water species *M. galloprovincialis* has proven an aggressive invader^8^,^9^. Similarly, lineages of the barnacle *Balanus glandula* from warmer southern areas of the native range in North America invaded similarly warm coasts of Argentina, while lineages from colder northern areas were found in similarly cold areas in Japan^10^. However, for most marine cases where cryptic invasion has been demonstrated, little is known of the ecological differences among the cryptic lineages.

The bryozoan genus *Watersipora* Neviani, 1895 is a promising system to investigate the importance of cryptic invasions. The Bryozoa are a phylum of encrusting animals that have become common in fouling communities the world over due to a predisposition for human-mediated transport^11^,^12^, either in ballast water^13^ or on ships' hulls^14^. Species of *Watersipora* are among the most invasive Bryozoa. Once released, populations of *Watersipora* grow explosively due to lateral growth of established colonies and settlement of short-lived larvae that may be retained near parents owing to short dispersal durations (generally <24 h)^15^,^16^. Introduced *Watersipora* have become a major space occupier on natural and anthropogenic substrata in protected bays in many areas^17^,^18^. In communities experimentally polluted with copper ions (an active agent in antifouling paint)^19^,^20^, or subjected to heat-wave conditions^21^, invasive *Watersipora* species have settled and occupied space more successfully than most indigenous organisms, suggesting adaptations favoring the rapid spread of these species.

Cryptic diversity in *Watersipora* appears rampant and is incompletely resolved^19^ genetic analysis is therefore critical to resolving introduction patterns. As currently understood, the genus consists of *W.* *arcuata* Banta, 1969, a species with distally recurved tentacle apertures^22^ and supported by monophyly of mitochondrial Cytochrome c oxidase subunit I (COI)^23^ sequences, and a group of genetically distinct clades we refer to as the *W. subtorquata-*complex that share a proximally pointed sinus in the lophophore aperture (henceforth “sinusoidal”). This latter group has a tortured taxonomic history that has been described as a taxonomic “can of worms”^24^. The taxonomic difficulty associated with this complex is largely because the genus lacks the spines, avicularia, and external ovicells used for taxonomic diagnosis in most other bryozoans.

The nominal species *Watersipora subtorquata* (d'Orbigny, 1852) is a widespread invader in cool-temperate areas globally. However, a divergent lineage (15% Kimura 2-parameter nucleotide divergence in COI) from the previously known *W. subtorquata-*complex was found as a single colony collected in California (this clade was referred to as *W.* “new sp.”^23^) and in subsequent sampling in California^25^. Thus, it appears at present that at least two cryptic species comprise the *W. subtorquata-*complex, and that genetic analysis is necessary to discover their patterns of distribution. While the type specimen locale of *W. subtorquata* is Rio De Janeiro, Brazil, native ranges of *W. subtorquata-*complex species are not known due to uncertainties of taxonomy and dispersal history, which may include undocumented movement by vessels. A third species, *W. subovoidea* (Ryland et al., 2009), which may be invasive in tropical areas, was recently re-described and formally separated from the *W. subtorquata-*complex following confirmation of genetic divergence in COI^26^. Ryland et al.^26^ postulate that *W. subovoidea* may be native to the Mediterranean, however origin is uncertain, and the IndoPacific presents another possible source for this species group according to suggested morphological affinity^27^.

Recognition of introductions of *Watersipora* began when it was realized that the species now referred to as *W. arcuata* and thought to be native to the tropical eastern Pacific^28^, had invaded Australian coastline. In fact, by the mid 20^th^ century, *W. arcuata* was extremely common around Australian harbors, indicating hull-fouling in spread^23^,^29^, but the arrival time can only be approximated to within 1889–1940 due to a dearth of intervening surveys^29^. An introduction of *Watersipora arcuata* to New Zealand occurred around 1957^30^ from which time populations spread to become common on the north and south islands^31^. It is thought *W. arcuata* invaded in southern California, near Los Angeles around 1961^28^. Colonies of a sinusoid species form, referred to generally as *W.* “*subtorquata*”, were recognized as invading in Australia in the 1970s and in New Zealand and California in the 1980s^31^,^32^. On the three landmasses, some regional displacement of *W. arcuata* was seen as colonies of sinusoid morph entered areas occupied by *W. arcuata*^17^,^31^. Indicating that the rapid propagation of sinusoid *Watersipora* colonies occurring along California coastline did not originate from simply one introduction, an initial study using COI sequence comparison located a “*subtorquata*” haplotype occurring in Australia and elsewhere, along with an example of COI sequence that was divergent (in Monterey Bay California), this genetic group being referred to as the “new sp.” COI phylogroup^23^.

Our first objective was to determine the distribution of the cryptic species noted above in California, and whether intensive sampling of these communities would indicate a likelihood that the number of sources in introductions is greater still than recognized. The genetic (COI) analysis was used secondly to investigate whether *Watersipora* species were non-randomly distributed with respect to sea surface temperature (SST) in the California region and globally. By contrasting multiple invasions of multiple species, our analysis suggests that different temperature-related physiological mechanisms may be important drivers of the invasive distributional patterns of the *Watersipora* lineages.

## Results

We generated 361 sequences (Table 1) with median length of 510 nucleotides and an average base content of A, 31.3%; T, 33.3%; C, 18.5%; G, 16.9%. Amino acid translations had no stop codons. Tajima's *D* statistic^33^ was a mean of −0.1795, ranging generally between 0 and 2 (Table 2). Despite slight negativity (as is typical^34^), few populations showed significant deviation from the simulated *D* null distribution, with the exception of some southern Californian *W. subtorquata* population samples, as discussed below.

**Figure 1:**
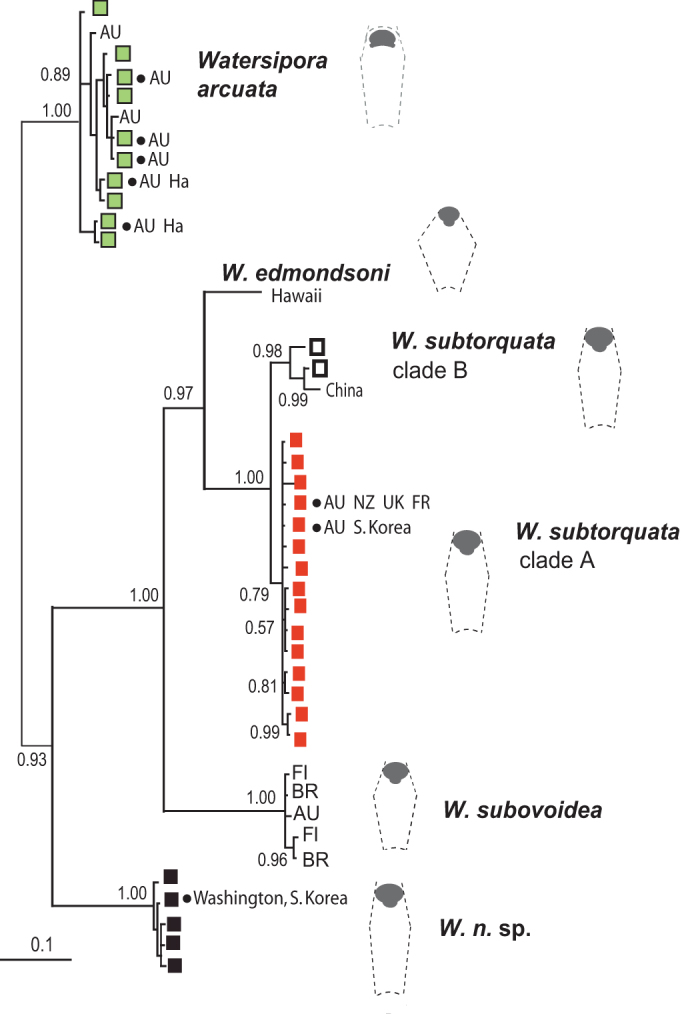
Bayesian tree of COI sequences. Posterior probabilities (> 0.5) are shown at nodes. Squares at branch tips, within four phylogroups – *Watersipora arcuata*, *W. subtorquata* clades A and B, and *W*. *n*. sp. – indicate an introduced haplotype on the Californian coast. Haplotypes found in other areas are indicated: Washington state (Pacific US); AU, Australia; Fl, Florida (Atlantic, US); BR, Brazil; Ha, Hawaii (O'ahu); NZ, New Zealand (Wellington); UK (southern England and Channel Islands); FR, France. *Watersipora* colonies have uniform zooids. Typical dimensions were determined from multiple colonies in different COI groups, providing a stereotyped zooid appearance (see Figure 4).

In California we found divergent COI clades that correspond to previously recognized *Watersipora arcuata*, *W*. *n*. sp. and *W. subtorquata* phylogroups. Sequences were also included in the Bayesian tree (Figure 1) from colonies identified as *W. edmondsoni* (on coral, n = 2, Kane'ohe Bay, Hawaii) and *W. subovoidea* colonies from southern Florida, Brazil, and tropical Australia (Table 1). Monophyletic groups differed by average net divergence of 18.5% (Kimura-2 parameter model); the lowest divergence was observed between *W. subtorquata* and *W. edmondsoni*, 12.3%; greatest divergence was observed between *W. arcuata* and *W*. *n*. sp. 24.0%). *Watersipora subtorquata* sequences differing by a net divergence of 2.8% formed clades we refer to as *W. subtorquata* clades A and B (Figure 1).

**Figure 2:**
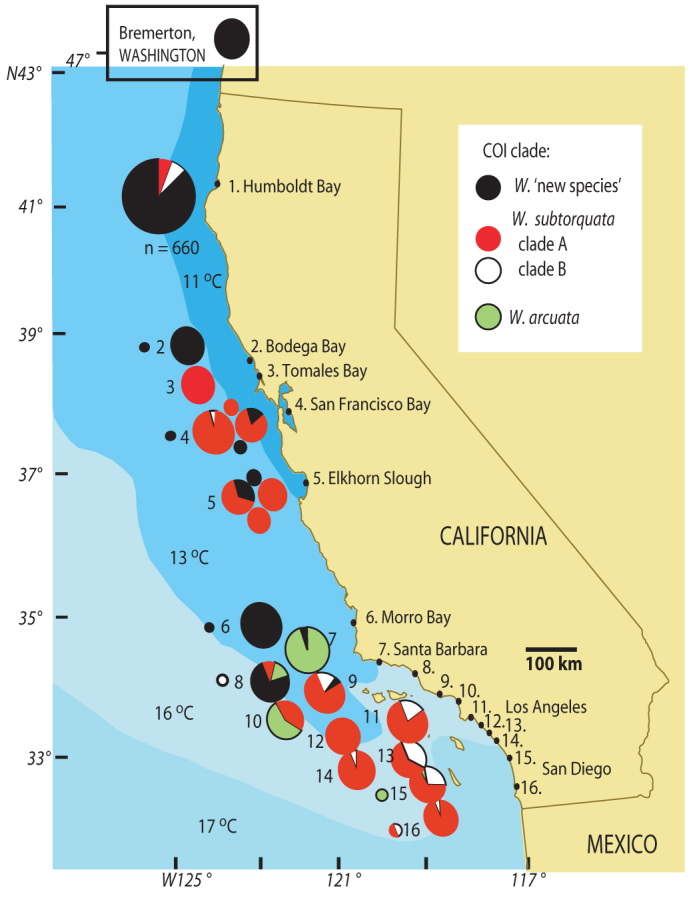
Distribution of *Watersipora* COI phylogroups in the California region, (regional mean annual sea surface temperature indicated). Colonies were collected from 2002–2011. Circles to the left of corresponding site numbers are previously reported COI samples^25^. Circles are sized in proportion to the number of colonies collected at each site, except as indicated for Humboldt Bay where phylogoups of a larger number of colonies were determined using multiplex PCR^35^. Each circle represents a different sample location or year of sampling. Number-only labels: Port Hueneme (8), Channel Island Dock, Oxnard (9), Marina del Rey (10), Long Beach Harbor (11), Huntington Harbor (12), Dana Point (13), Newport Harbor (14), Oceanside (15), Mission Bay (16).

*W. subtorquata* clade A was abundant in southern California and in central California –Moss Landing, San Francisco and Tomales Bay (Figure 2). Clade B was common in southern California and was established at Humboldt Bay, northern California, where it was found in specimens collected in 2002 along with specimens of *n*. sp. clade. To increase our understanding of clade composition of Humboldt Bay, California we then determined COI phylogroup using a multiplex assay, in which five PCR primers each mismatched at its 3′ end to one or two *A, B*, or *new* sp sequence populations, generating phylogroup-specific fragment lengths viewed on agarose gels^35^. *W*. *n*. sp. was dominant at Humboldt Bay (92.67%), with clade A (3.84%) and clade B (3.49%) being both present at lower frequency (Figure 2).

**Figure 3:**
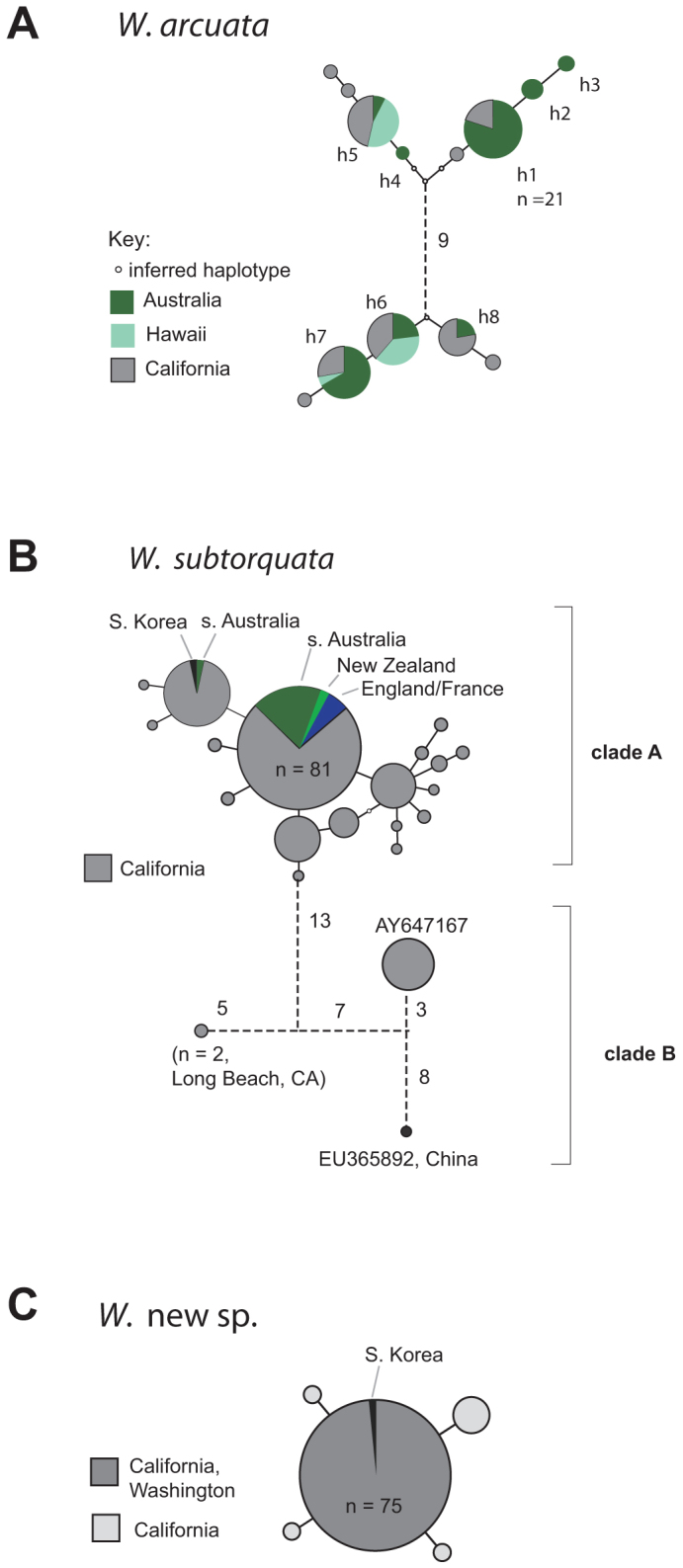
Parsimony networks describing relationships of COI haplotypes of *Watersipora*, introduced to California and other areas. Continuous straight lines represent connections with >95% confidence^68^ and dashed lines represent connections with confidence below this limit. Circles indicating sampled haplotypes are scaled according to haplotype frequency. The frequency of the most common haplotype in each set is shown. *W. arcuata* haplotypes, h1–h8, were defined previously^23^.

*W. subtorquata* clade A has been the most common group sampled at a global scale to date (Figure 3). The most common clade A sequence in California (referred to as haplotype *WS1*^23^) is also common in southern Australia, New Zealand, and Europe (Figure 3B). The second most frequent haplotype of clade A (*WS3*) occurs also in southern Australia and South Korea (Figure 1). Clade B variation consisted of one haplotype characterized previously^25^ (GenBank accession: AY647167) and a second haplotype represented by two colonies collected at Long Beach (near Los Angeles), and a third unique sequence represented by a single colony sampled previously in China^34^ (collected on seaweed at Qingdao Huiquan Beach, Qingdao, GenBank accession: EU365892) (Figure 3B).

The *W*. *n*. sp. haplotypes, consisting of one common sequence and several less frequent, related haplotypes (Figure 3C) were common at many California sites. The southernmost finding of *W*. *n*. sp. was Oxnard, central California, which is within the region defined by the 16°C long term mean SST isotherm (Figure 2). *W*. *n*. sp. was the one group found at Bodega Bay, Morro Bay, and Bremerton. Clade A and *n*. sp. occurred at similar frequencies in the area of San Francisco and Monterey Bay.

The five COI phylogroups that we recognized occur in statistically distinguishable SST regimes according to a partial Mantel test that included all five groups (*P* < 0.0001). From lower to higher SST, these were *W*. *n*. sp. (median = 11.8°C, 95% confidence interval 10.9−15.1°C, n = 111), *W. subtorquata* clade A (15.1°C, 14.0−17.3°C, n = 212), *W. arcuata* (16.3°C, 15.1−25.7°C, n = 82), *W. subtorquata* clade B (17.1°C, 11.8−18.2°C, n = 29), and *W. subovoidea* (26.0°C, 25.0−26.3°C, n = 23). Partial Mantel test comparisons of SST for pairs of phylogroups produced significant *r* coefficients at *P*-values between 0.020 and 0.001 (statistically significant at an unadjusted α value of 0.05); the weakest correlation (r = 0.1008) occurred in comparison of *W. arcuata* and *W. subtorquata* clade A distributions (*P* = 0.0127).

Population pairwise Φ_ST_ measures were generally higher in the *W. subtorquata* clade A, B and *arcuata* samples than among *n*. sp. populations (Table 2). Given the fact that the *W*. *n*. sp. had relatively low nucleotide diversity, COI is likely insufficiently sensitive for detecting post-introduction genetic isolation if present. However, there was significant differentiation (Φ_ST_) occurring between the Morro Bay sample and other populations (pairwise comparisons of P < 0.05), but no other significant differentiation.

Ten *Watersipora arcuata* haplotypes were found in California (Figure 3A). Two (previously designated *h1* and *h8*, Mackie et al. 2006) were identical to haplotypes previously found in Australia, and three (*h5*, *h6*, and *h7*) identical to haplotypes known from both Australia and O'ahu, Hawaii. The COI variation of *W. arcuata* showed structuring at the scale of collection sites (Φ_ST_, = 0.4459, *P* < 0.0001) and collection areas (i.e., within O'ahu, California, Perth or Adelaide; Φ_SC_ = 0.6285, *P* <0.0001). There was, however, no differentiation observed in comparing the regional sampling areas of O'ahu, California, Perth or Adelaide. In fact, the inter-area variance component was negative (Φ_CT _ = −0.4912), indicating a spatially-dispersed but locally-structured pattern.

Spatial differentiation was evident within the *W. subtorquata* COI clade complex found on the California coastline. An AMOVA supported differentiation of northern and southern populations (from Santa Barbara southward) (Φ_CT_ = 0.1068, *P =* 0.0215); these populations were also structured in COI nucleotide variation at local scale (Φ_ST_ = 0.1625, *P* < 0.0001, Φ_SC_ = 0.06229, *P* = 0.0401). It is noted that southern Californian populations have *D* statistic deviations that may be due to the sampling of relatively long branches (clade A and B). Newport and Mission Bay (sites 14 and 16, Figure 2) had *D* statistic^33^ values of < 2.0, which were statistically significant (*P_D_*_ obs > *D* exp_ < 0.05); this appears to reflect unbalanced frequencies of haplotypes separated on divergent branches. In contrast, Dana Point (*D* = +2.388, *P_D_*_ obs > *D* exp_ = 0.9989), and Oceanside (*D* = 0.5516, *ns*) had positive *D* measures, reflecting the more even spread of clades A and B in these samples.

**Figure 4:**
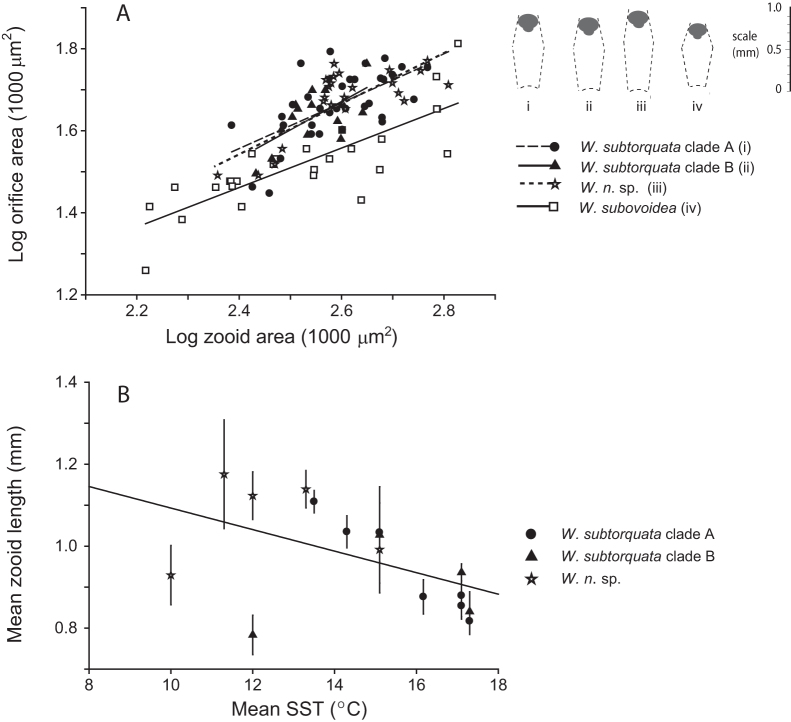
(A) Averaged orifice area versus zooid area (log_10_ versus log_10_) for populations distinguishable as four COI clades. As shown previously^26^ the plotted relationship discriminates the recognized species *W. subtorquata* and *W. subovoidea*, however as evident here, it does not discriminate the *subtorquata* (A or B) and *n.* sp. phylogroups. Inset: cartoon of zooids of averaged proportions, by COI phylogroup. (B) Plot showing means and SE (vertical bars) of zooid length at different sites on US west Coast, indicating a decrease in length toward warmer localities. Trend lines were calculated by least-squares regression.

The zooid area-to-operculum area ratio (Figure 4A) distinguished *Watersipora subovoidea* colonies as a homogeneous grouping from other sinusoidal colonies consisting of *subtorquata* and *n*. sp. COI clade colonies (ANCOVA: *F*_1_,_97_ = 59.83, *P* < 0.0001). An ANCOVA, comparing morphometric ratio slopes did not support a difference between *W. subtorquata* (clade A or B) and *W*. *n*. sp. populations (*F*_1_,_70_ = 0.18, *P* = 0.6727). In considering the invasive *subtorquata*–*n* sp. complex in California, there was no relationship between the COI-clade identity and zooid length (data not shown), however, zooid length scaled with the mean SST temperature in the invaded area (Figure 4B). Deviating from the general trend, zooid lengths of *n.* sp. colonies collected from Bremerton, Washington (mean SST ~ 10°C, collected in 2010), and *W. subtorquata* clade A colonies from Humboldt Bay (mean SST ~ 10°C, collected in 2003) were short, for reasons unknown. Analysis of the general sample of sinusoidal *Watersipora* colony populations in California supported a negative relationship between temperature and zooid length (*R*^2^ = 0.245, *P* < 0.001) (Figure 4B).

## Discussion

On the basis of phylogenetic inference using the COI locus, the previously described *Watersipora subtorquata*-complex represents two cryptic species, *W. subtorquata* and *W*. *n*. sp., consistent with previous reports^23^. According to the present sampling, *W. subtorquata* can be further divided into two genetically shallow groups − clade A, which has haplotypes recognized in Europe and Australasia, and clade B, found to be common in southern California and present at much lower frequency in northern California. Other investigations show that these three known COI clades of the *W. subtorquata*-complex also occur in the Asian western Pacific^36^,^37^. The absence of reports of sinusoidal *Watersipora* in Californian waters prior to the 1980s probably indicates the true absence of such forms given the intensity of study there^32^. Since then, according to COI data, there have been multiple introductions from separate sources. The introduced *Watersipora* of the Californian region is more diverse than that of Australasia, and COI variation apparent in California reflects either different thermal tolerances of the *W*. *n*. sp. and *W. subtorquata* complex source populations, different sets of introductions to these areas, or both.

*Watersipora* species as a whole were absent from the US Pacific coastline until the 1960s and absent from the fossil record in that region^28^. *Watersipora arcuata* was the first watersiporid to be recognized on the coastline, appearing in southern California around 1963^28^. Soule and Soule^32^ then reported a sinusoid species as invading in Los Angeles Harbors and marinas in 1982–3, a period of unusually warm water due to El Niño. We have no reason to suspect that these specimens were not the sinusoidal *Watersipora* *subtorquata*-complex described here. Examination of collections of the late Dorothy and John Soule at the Santa Barbara Natural History Museum unfortunately did not locate the material referred to in their report (Mackie, pers. obs., July, 2011). Banta^22^ suggested *W. arcuata* was native to the tropics or subtropics of the eastern Pacific. Evidence of the native source of *Watersipora* *subtorquata* clade A and clade B or *W*. *n*. sp. is lacking, as is a precise timing of arrival in California.

Early collections of colonies that best fit the morphological description of *W. arcuata* were made in the Galapagos Islands and the Pacific Mexican coast, including Baja California and Gulf of California, prior to the 1930s^28^,^38^. By 1940, *W. arcuata* was a common fouling species in the Gulf of California^39^,^40^ but was not yet known on the US Pacific coastline. Two hypotheses have been proposed to explain the appearance of *W. arcuata* in California in the 1960s. Soule and Soule^32^ proposed that larval dispersal or dispersal of colonies on drift material from warmer Pacific areas into California could occur during El Niño events (such as 1956–57), when currents from southern areas extend further north than usual. Banta and Carlton each, however, favored a scenario of introduction of *W. arcuata* to California from Australasia through ship fouling, in part based on negative evidence, specifically the lack of *W. arcuata* in extant fouling community records or the fossil record of California^32^. Positive circumstantial evidence includes the observation that Australasia has been a donor of a number of marine invasive species to the US Pacific region^41^.

Given the lack of segregation of COI genetic variation among widespread areas – Australia, California, and Hawaii – the arrival of *W. arcuata* colonizers through shipping from common sources is supported, as opposed to regionally independent introductions. Defining the routes of introductions around the globe based on COI is not possible though given the observed distribution of genetic variation at that locus. Inferring direction and chronology of these invasions genetically will likely rely on the use of multiple loci providing finer spatial resolution to the distribution of genetic variation.

*Watersipora* species ranges have undergone remarkable shifts with human assistance. Given such widespread global introductions and realizing that ranges are rapidly dynamic, source populations are perhaps traceable now only by searching for remnant phylogeographic patterns.

Collection records of *W. arcuata* and *W. subtorquata* indicate that species range boundaries have changed rapidly. In the 1980s *W. subtorquata* replaced *W. arcuata* in southeastern Australia and New Zealand, specifically in cooler areas of these landmasses^17^,^31^. In the areas of *subtorquata* introduction, *W. arcuata* had been present since its introduction sometime between the late 1800s and 1940 in Australia^29^ and around 1957 in New Zealand^30^. These taxonomic records indicate that species of Watersipora may compete with one another for resources (with space presumably being one of the most important) in the human-modified fouling niche. Further population separation on the basis of COI supports widespread species-replacement interactions.

In the present study we confirmed that *W. subovoidea* (matching northern Australian and Brazilian populations morphologically) also occurs in Florida, US. COI sequences show ≤1% divergence among *W. subovoidea* in Brazil, Florida and Australian populations, supporting recent and widespread introductions of a species suited to tropical conditions. While genetic analysis of the type specimens or neotypes has not been conducted, a *W. subtorquata-W. subovoidea* delineation is supported on the basis of both morphometric and COI comparison^26^. The *W. subtorquata* holotype (Gunabara Bay, Brazil) was described by d'Orbigny from material collected in 1837. Given this historical record, it was surprising that recent collections in the Rio De Janeiro and Sao Paulo regions have revealed only *W. subovoidea*. Similarly Ramalho et al.^26^ reported collections of *Watersipora* matching *W. subovoidea* morphologically at multiple localities spanning a wide range of surfaces and pollutant levels. Thus, there is reason to suspect *W. subovoidea* has displaced *W. subtorquata* in its native locale or at least an area where it was common in the 1800s, and there is clearly a need to relate collections from different points to the diversity patterns now suggested by genetics.

Prior studies of Bryozoa have suggested genetically divergent species are recognizable by morphological divergence^43^,^44^, but our analysis suggests that this conclusion is not universal. Measurements of subsamples of *W*. *subtorquata* clades A and B and *W*. *n*. sp. in California showed these to be homogeneous in their zooid geometry (Figure 4A) and they were not definitively sorted by color (unpublished data). Colonies of *W*. *n*. sp. varied from flat, encrusting forms to large (30 cm) multi-colony ball-shaped forms. Although diagnostic morphological criteria may yet be discovered, currently none seem present that would be practical for rapid identification, and genetic analysis will be necessary for continued work on *Watersipora*.

The lack of morphological distinctiveness of *W. subtorquata* and *W*. *n*. sp. COI clades reflects an absence of obvious skeletal characters (a situation that is exacerbated by the relatively featureless zooid morphology of the genus), or wholesale hybridization leading to a continuous range of morphologies. Hybridization is a factor affecting identification and ecological responses in introductions of the *Mytilus* species complex, for example^9^. No introgression of mitochondrial DNA has been found in colonies of the *W. arcuata* and *W.* *subtorquata* morphologies (arcuate and sinusoidal orifice form respectively), which supports the standpoint that *Watersipora* COI clade groups are not randomly interbreeding. Assessment of genetic variation at microsatellite and other nuclear DNA loci is being used to test for admixture among COI clade populations.

Zooids were as much as 25% longer in northern California as compared to southern Californian colonies. The phylogroup itself showed no consistent relationship to zooid length, with rather, colony populations of multiple groups exhibiting latitude related zooid length (Figure 4). Size-latitude trends arguably still require extended documentation in invertebrates generally; in mollusks a recent meta-study has however indicated size trends to be common, with the direction of the relationship being variable^45^. The direction of the zooid size to temperature relationship seen across these recently introduced *Watersipora* populations *is* consistent with studies of other *Watersipora* spanning the Galapagos archipelago^45^ and in other bryozoans where there is an inverse relationship between zooid size and temperature^46^,^47^,^48^. Phenotypic plasticity is a possible explanation (as seen in one study of a limpet in which shell variation - larger size in cold - was explainable by water temperature rather than genetic variance^49^). *Drosophila* wing-traits^50^ on the other hand provide an example of post-introduction variation responding in a clinal selective gradient. There are sharp differences in mean size are recognized following introductions in a number of marine metazoans^51^. Perhaps promisingly, *Wateripora* provide a useful system in which to assess the heritable/plastic components of zooid size, along with net overall growth, and reproductive characteristics, determining whether these variously influence or respond to observed range expansions.

The boundary presented in part by the cold California and warmer Davis current systems allows the California coast to be used as a sensitive test of the role of temperature in differentiating introduction processes. Examination of COI variation occurring in *Watersipora* revealed significant north-south separation of haplotype frequencies in California. This separation coincides with Point Conception, an area with a high turnover of ranges and phylogeographic breaks in native taxa^52^. All *W. arcuata* occurrence was to the south of Point Conception (and *n*. sp., conversely, has not been found far south of Point Conception). The range of *W. arcuata* however did expand briefly in a northward direction in 1982 and 1983, an El Niño period, such that the species was found in Monterey Bay, northern California^32^ where it has not been reported subsequently. This particular observation is notable for indicating the likely sensitivity of the ranges to temperature. Our study, and others examining genotypic variance (e.g^10^,^53^,^54^,^55^), suggest genetically related invading propagules have temperature related fitness which determines organismal or genotype-level range limits at least in early stages of introduction. Average sea-surface temperatures of 14°C–20°C unite the southern Californian and some Australian localities, where *W. arcuata* and *W. subtorquata* were found together. The situation is analogous to invasions of two monophyletic *Caulerpa* (Chlorophyceae) groups^56^,^57^, which also appear to be established in southern Australia, Mediterranean areas and southern Californian regions, but not northern California.

While the COI phylogroup-SST correlation is derived from relatively few global locales, it is possible to define widespread introductions of the five *Watersipora* COI groups by different temperature-zone envelopes, an indication that intrinsic differences in temperature-related fitness structure patterns of spread. With this background information, direct hypothesis testing can be used to determine whether phenotypic differences as opposed to vector transport patterns alone dictate introduction success or local densities of *Watersipora* species. The COI variation of California (which is greater than that observed in Australia) and haplotype distribution pattern provides a useful framework for common-environment experiments to test for physiological restrictions to ranges. The association of different species and COI clades of *Watersipora* with particular temperature zones suggests a global assortment of lineages into similar temperature zones, in other words, natural selection acting on existing variation in parallel in different areas.

While the invasion success of *Watersipora* populations is geographically limited by evolved ecological tolerances, the species in the genus as a whole have cumulatively extremely broad potential for global spread, with a collection of traits (including a high tolerance of copper-based antifouling paint that is apparent in larvae and colonies) which assists in colonization of painted hulls on ships that may further transport colonies. Perhaps because growth rate and reproductive potential are intrinsically connected in modular organisms, temperature modulated growth rate (temperature-related fitness) may prove more useful than other general hypotheses commonly put forward to explain the high invasive capacity of certain introduced species, such as the ability to escape specialist predators and pathogens^58^,^59^,^60^ or propagule pressure^61^,^62^. Clearly, modular fouling organisms warrant attention as a group of organisms sensitively indicating community changes in response to environmental change.

## Methods

### Collections

Colonies were collected between 2005 and 2010 from docks and floats, predominantly, throughout California, and additional specimens were obtained from fouling panels in San Francisco Bay, and Humboldt Harbor (collections made in the period of 2002–2005), and field collections from Washington State (Bremerton), Florida, and Brazil (Tables 1 and 2). We generated COI sequence for 361 colonies. Sequences and collection information were lodged in GenBank (accession numbers: JQ715456–JQ715577). We included previously reported sequences^23^,^25^,^26^,^36^,^37^ in analyses.

### PCR and sequencing

Colonies were preserved in 85–95% ethanol. Fragments (which were generally <2 cm across) were sorted into individual colonies with independent ancestrula. DNA was extracted by Qiagen DNeasy Tissue protocol. DNA for sequencing was obtained by amplification of 710 base pairs using LCO1490 and HCO2198 primers^63^, followed by re-amplification of this product in a second PCR with LCO1490 and a bryozoan-specific primer BRY-HCOI-2161, effectively increasing product yield^23^. PCR was carried out using GoTAQ® DNA polymerase and 2x Buffer, with 3.0 mM Mg^2+^ ion, at an annealing temperature of 40°C. Products were isolated using Qiagen Quickspin® columns and sequenced in both directions by BigDye® di-deoxy terminators.

### Sequence analysis

Sequence chromatograms were read using Codon Code Aligner ® software, aligned using MEGA4^64^, and collated to haplotypes using DNA Collapser V. 1 (http://www.birc.au.dk/fabox). A Bayesian analysis (using a flat prior distribution and a General Time Reversible model of nucleotide substitution including a Gamma-distribution substitution rate parameter) was used to construct a tree. Nucleotide substitution model parameters were determined using ModelTest^65^, and the tree constructed using MyBayes^66^. Posterior probabilities at nodes were calculated using three parallel Metropolis Coupled Markov Chains, searching for 2 million generations. The Bayesian analysis produced a robust topology with posterior support for major clades of 0.94 or higher, at which level there was topological agreement with a parsimony tree found by heuristic search in PAUP*^67^ (data not shown).

Haplotype relationships within *Watersipora arcuata, W*. *subtorquata* and the *W*. *n*. sp. clades were evaluated using median joining parsimony networks^68^. Sequence lengths used in comparisons were determined by the minimum lengths of sequence data available in GenBank: a 388-nucleotide segment of *W. arcuata* COI sequences was compared, including samples from southern Australia (Perth, Adelaide, Sydney areas) and O'ahu, Hawaii^23^ *Watersipora subtorquata* and *W*. *n*. sp. clade networks were constructed using a 489-nucleotide segment. AMOVA (Analysis of Molecular Variance^69^) was used to quantify COI sequence variation partitioned among broad geographic regions for both *W. arcuata* (regions included Hawaii, California, and Australia) and *W. subtorquata* (including the Australasian region of southern Australian and New Zealand and two California regions north and south of Point Conception). Permutation tests (5000 replicates) were used to evaluate AMOVA coefficient significance^70^.

### Mean sea surface temperature (SST) approximation

Local average SST was approximated using year-long measurements spanning 2002–2011. For most US sites, SST was obtained from a monitoring buoy located within 50-km of the sampling location, via NOAA National Oceanographic Data Center Coastal temperature tables (http://www.nodc.noaa.gov/dsdt/cwtg/cpac.html). Measurements were also obtained using the NASA satellite (Aqua) Moderate Resolution Imaging Spectroradiometer (MODIS) thermal map data archive^71^. Temperatures were resolved to 1°C unit of accuracy using the dominant pixel-record within 50×50 km squares positioned offshore to sampling areas. We verified, as elsewhere^72^, that MODIS and buoy-recorded mean SSTs were generally within 1°C.

### Analysis of SST data

The 95% confidence interval of the median sea surface temperature experienced by major COI clades was estimated by bootstrapping (resampling populations of 20 individuals for 1000 replicates). To test the null expectation of no correlation between temperature and clade, Mantel tests were conducted correcting for spatial distance using a partial matrix^73^. Pairwise SSTs and decimal grid coordinates were converted to Euclidean distances. Clades were encoded as presence or absence, and separate tests were run for all five clade groups and pairs of clades separately. Partial Mantel tests were conducted using the *R* Software Project package, ecodist^74^. Data were ranked, which assists in linearizing relationships between dissimilarity matrices^75^. The significance of the partial coefficient was determined using 10,000 matrix permutations.

### Zooid-dimension comparisons for COI clades of sinusoidal *Watersipora*

The recognized species *Watersipora subovoidea* and the *W. subtorquata-*complex can be distinguished by zooid proportions: for a given frontal shield area, the tentacular orifice is smaller in *W. subovoidea*^26^. In the current study, a subset of the sinusoid colonies analyzed by COI were photographed at 20X magnification using a dissecting scope, and analyzed using Image J imaging software^76^. We recorded five zooid dimensions: zooid length (*L*_z_), zooid width at maximum (*W*_z_), orifice width (*W*_or_), orifice length (*L*_or_). We tested for a difference between *W*. *n*. sp. and other *W. subtorquata-*complex COI phylogroups, using zooid area and tentacular orifice area (as in^26^) as covariates via ANCOVA. Log_10 _transformations of areas were used, and regressions met the assumption of homogeneity of variances.

## Author Contributions

All authors contributed to experiment design, experiments, manuscript text, and reviewed the manuscript. Mackie prepared figures.

## Figures and Tables

**Table 1:** Specimens analyzed, listed according to phylogroup, and sample location along with estimated mean annual sea surface temperature (SST)

Region	Site or Area name	Coordinates^A^ (Lat., Long.)	mean SST (°C)	COI phylogroup	*N*	Source (if another study)
w. US	Bremerton	47.5798, −122.6321	10.0	*n.* sp.	12	
w. US	Bodega Bay	38.00 , −123.00	11.3	*n.* sp.	1	**25**
w. US	Bodega Bay Harbor	38.3295, −123.0562	11.3	*n.* sp.	16	
w. US	Humboldt Harbor	40.8074, −124.1635	11.8	B	11	
w. US	Humboldt Harbor	40.8074, −124.1635	11.8	*n.* sp.	33	
Europe	Plymouth	50.30 , −4.14	13.0	A	1	**26**
Europe	Guernsey	49.50 , −2.58	13.0	A	2	**26**
Europe	St Jacut	48.60 , −2.15	13.0	A	4	**26**
w. US	Moss Landing Harbor	36.8051, −121.7852	13.0	A	25	
w. US	Moss Landing Harbor	36.8051, −121.7852	13.0	*n.* sp.	12	
w. US	Morro Bay	35.37, −120.86	13.3	*n.* sp.	1	**25**
w. US	Morro Bay	35.3707, −120.8585	13.3	*n.* sp.	14	
w. US	San Francisco Bay	37.91, −122.35	13.5	*n.* sp.	1	**25**
w. US	San Francisco, Richmond	37.9130, −122.3503	13.5	A	25	
w. US	San Francisco, Richmond	37.9130, −122.3503	13.5	B	1	
w. US	San Francisco, Richmond	37.9130, −122.3503	13.5	*n.* sp.	1	
w. US	San Francisco, Oakland	37.8102, −122.3230	13.5	A	1	
w. US	San Francisco, Oakland	37.7845, −122.2676	13.5	A	4	
Australia	Hobart	−43.00, 147.28	14.0	A	2	**23**
Europe	Wellington	−41.00 , 174.78	14.2	A	4	**23**
w. US	Tomales Bay	38.1991, −122.9220	14.3	A	17	
w. US	Ventura	34.17, −119.23	15.1	B	1	**25**
Australia	Melbourne	−38.00, 144.82	15.1	A	7	**23**
w. US	Channel Islands Harbor	34.1666, −119.2250	15.1	A	13	
w. US	Channel Islands Harbor	34.166 , −119.2250	15.1	*n.* sp.	1	
w. US	Channel Islands Harbor	34.1666, −119.2250	15.1	B	3	
w. US	Port Hueneme	34.1532, −119.2095	15.1	arcuata	3	
w. US	Port Hueneme	34.1532, −119.2095	15.1	A	2	
w. US	Port Hueneme	34.1532, −119.2095	15.1	*n.* sp.	14	
w. US	Marina Del Rey	33.9702, −118.4496	15.1	arcuata	8	
w. US	Marina Del Rey	33.9702, −118.4496	15.1	A	3	
w. US	Santa Barbara	34.4067, −119.6890	16.0	arcuata	19	
w. US	Santa Barbara	34.4067, −119.6890	16.0	*n.* sp.	1	
Australia	Adelaide	−34.50, 138.53	16.3	arcuata	12	**23**
Australia	Adelaide	−34.50 , 138.53	16.3	A	1	**23**
w. US	Oceanside	33.21, −117.40	17.1	arcuata	2	**25**
w. US	San Diego, Shelter Island	32.71, −117.23	17.1	A	1	**25**
w. US	Oceanside	33.2121, −117.3954	17.1	arcuata	1	
w. US	Oceanside	33.2121, −117.3954	17.1	A	12	
w. US	Oceanside	33.2121, −117.3954	17.1	B	3	
w. US	Mission Bay	32.7671, −117.2362	17.1	A	18	
w. US	Mission Bay	32.7671, −117.2362	17.1	B	1	
w. US	Long Beach Harbor	33.7655, −118.2528	17.3	A	17	
w. US	Long Beach Harbor	33.7655, −118.2528	17.3	B	4	
w. US	Huntington Harbor	33.7175, −118.0658	17.3	A	17	
w. US	Dana Point Harbor	33.4591, −117.6992	17.4	A	8	
w. US	Dana Point Harbor	33.4591, −117.6992	17.4	B	5	
w. US	Dana Point, Tijuana Est.	33.4614, −117.7146	17.5	A	3	
w. US	Newport	33.6199, −117.8943	18.2	A	14	
w. US	Newport	33.6199,−117.8943	18.2	B	1	
n. Asia	Korea, Namhae Sangju	34.71, 127.99	20.0	A	1	**37**
n. Asia	Korea, Namhae Sangju	34.71, 127.99	20.0	*n.* sp.	1	**37**
Australia	Sydney	−33.87 , 151.21	20.3	arcuata	17	**23**
Australia	Sydney	−33.87 , 151.21	20.3	A	6	**23**
Australia	Perth	−31.93 , 115.83	20.5	arcuata	10	**23**
Australia	Perth	−31.93 , 115.83	20.5	A	3	**23**
n. Asia	Qingdao	36.054 , 120.38	23.0	B	1	**36**
e. US	Florida	27.20, −80.22	25.0	subovoidea	4	**23**
Europe	O'ahu	21.00, −157.87	25.7	arcuata	12	**23**
Australia	Dampier	−20.66 , 116.71	26.0	subovoidea	4	**23**
Brazil	Rio De Janeiro	−23.81, −45.43	26.0	subovoidea	14	
Australia	Cairns	−16.88, 145.80	26.5	subovoidea	1	**23**

^A^ Coordinates given to four decimal places refer to sampling site from present study. Coordinates to two places refer to regional-level locality information derived from other sources.

**Table 2:** Summary of population diversity and pairwise Φ_ST_ measures for *Watersipora arcuata*, *subtorquata*, and ‘new sp’. COI sequences (length: 489 base pairs)

**Diversity indices *Φ*_ST_**																
**A. *Watersipora arcuata***																
	*n*	*K*	*S*	*S/n*	π	*D*		Santa Barbara								
Santa Barbara	19	8	18	0.9474	0.0168±0.0094	0.5843	Santa Barb.	−								
Marina Del Rae	7	4	14	2.0000	0.0123±0.0079	−1.3759	Msr. Del R.	0.8557***								

**B.** ***Watersipora subtorquata***																
	*n*	*K*	*S*	*S/n*	π	*D*	Aust-NZ	Tomales Bay	San Fran. Bay	Elkhorn Sl.	Oxnard	Long B.	Hunt. Harb.	Dana Point	Newport	Ocean.
Australia-New Zealand^A^	23	2	1	0.0435	0.0002±0.0004	−1.1610	–									
Tomales Bay	17	5	4	0.2353	0.0018±0.0015	−0.7400	0.2039***	–								
San Francisco Harbor^B^	24	7	7	0.2917	0.0037±0.0024	−0.1351	0.3967***	0.0785*	–							
Elkhorn Sl.	19	7	7	0.3684	0.0025±0.0019	−0.9214	0.4535***	0.0475	~ 0	–						
Oxnard	18	5	26	1.4444	0.0161±0.0088	0.1633	0.1752**	0.1082	0.1186*	0.1041*	−					
Long Beach, LA	20	7	32	1.6000	0.0175±0.0094	−0.2038	0.1576***	0.1030*	0.1208**	0.1045*	~ 0	−				
Huntington Harbor	15	5	6	0.4000	0.0036±0.0025	−0.1401	0.2290**	0.1713*	0.1460**	0.1649*	0.0907	0.0714	−			
Dana Point	13	5	25	1.9231	0.0255±0.0139	2.3880**	0.7302***	0.5128**	0.5996**	0.5200**	0.0508	0.0712	0.4669*	−		
Newport	14	4	24	1.7900	0.0082±0.0049	−2.0674**	0.0431*	0.0369**	0.0942**	0.0848*	~ 0	~ 0	0.0262	0.2158*	−	
Oceanside	15	4	24	1.6000	0.0171±0.0094	0.5516	0.2525**	0.1510*	0.1738*	0.1523*	~ 0	~ 0	0.1422*	0.0103	0.0051	−
Mission Bay	18	5	25	1.3889	0.0070±0.0042	−2.0950**	0.0726**	0.0601*	0.0586*	0.0799**	0.0035	0.0131	0.003	0.2916*	~ 0	0.0316

**C.** ***Watersipora new sp.***																
	*n*	*K*	*S*	*S/n*	π	*D*	Bremerton	Humb. (2003)	Humb. (2006)	Bodega Bay	Elkhorn Sl.	Morro Bay				
Bremerton (2010)^C^	12	1	0	0	0	0	–									
Humboldt Bay (2003)^B^	10	1	0	0	0	0	~ 0	–								
Humboldt Bay	17	2	1	0.0588	0.0002±0.0004	0.0058	~ 0	~ 0	–							
Bodega Bay	16	1	0	0	0	0	0	~ 0	0.0166	–						
Elkhorn Slough	10	1	0	0	0	0	0	~ 0	~ 0	~ 0	–					
Morro Bay	11	3	2	0.1818	0.0010±0.0011	−0.7782	0.1538*	0.1264*	0.0767	0.1905**	0.1318**	–				
Port Hueneme	13	3	2	0.1538	0.0006±0.0008	−1.4680	~ 0	~ 0	~ 0	0.0099	~ 0	0.0965				

Permutation tests of *D* and Φ_ST_ were conducted with 5,000 replicates.

*:*P* < 0.05.

**: *P* < 0.001.

***: *P* < 0.0001.

Unless indicated, samples were collected in California Department of Fish and Game (Introduced Species Surveys), 2006.

^A^Data were reported in Ref.23.

^B^Colonies were collected in Smithsonian (Environmental Research Center) surveys.

^C^Colonies were collected by Greg Jensen, University of Washington, 2010.
